# Genome characterization of Long Island tick rhabdovirus, a new virus identified in *Amblyomma americanum* ticks

**DOI:** 10.1186/1743-422X-11-26

**Published:** 2014-02-11

**Authors:** Rafal Tokarz, Stephen Sameroff, Maria Sanchez Leon, Komal Jain, W Ian Lipkin

**Affiliations:** 1 Center for Infection and Immunity, Mailman School of Public Health, Columbia University, 722 West 168th Street, Room 1701, New York, NY 10032, USA

**Keywords:** Ticks, Rhabdovirus, High-throughput sequencing, *Amblyomma americanum*

## Abstract

**Background:**

Ticks are implicated as hosts to a wide range of animal and human pathogens. The full range of microbes harbored by ticks has not yet been fully explored.

**Methods:**

As part of a viral surveillance and discovery project in arthropods, we used unbiased high-throughput sequencing to examine viromes of ticks collected on Long Island, New York in 2013.

**Results:**

We detected and sequenced the complete genome of a novel rhabdovirus originating from a pool of *Amblyomma americanum* ticks. This virus, which we provisionally name Long Island tick rhabdovirus, is distantly related to Moussa virus from Africa.

**Conclusions:**

The Long Island tick rhabdovirus may represent a novel species within family *Rhabdoviridae*.

## Background

The family *Rhabdoviridae* consists of a large group of enveloped, single-stranded, negative sense RNA viruses that infect a wide range of vertebrates, invertebrates, and plants [1]. Their genome typically consists of at least five open reading frames (ORFs) organized in a linear order 3′-N-P-M-G-L-5′, and encode the viral nucleocapsid (N), phosophoprotein (P), matrixprotein (M), glycoprotein (G) and RNA polymerase (L). In addition to these genes, many rhabdoviruses contain smaller ORFs that encode additional accessory proteins, most without known function. Currently, *Rhabdoviridae* consists of nine named genera (*Cytorhabdovirus, Ephemerovirus, Lyssavirus, Novirhabdovirus, Nucleorhabdovirus, Perhabdovirus, Sigmavirus, Tibrovirus, Vesiculovirus*), although many tentative rhabdoviruses still await taxonomic classification [2].

Arthropods are essential in transmission of many pathogenic rhabdoviruses. In the context of a program in viral surveillance and discovery in arthropods, we examined viromes of ticks collected in New York State and identified a novel rhabdovirus associated with the lone star tick, *Amblyomma americanum*. We provisionally name this virus Long Island tick rhabdovirus.

## Results

Analysis of high-throughput sequencing (HTS) data by BLASTx revealed sequences with homology to all five prototypical rhabdovirus proteins. Homology searches indicated these sequences were most similar to Moussa virus (MOUV) and therefore all were assembled to MOUV as a reference genome [3]. Amino acid analysis of the coding sequence suggested this virus likely represented a novel rhabdovirus; hence, we tentatively named it Long Island tick rhabdovirus (LITRV), after its geographical location and host.

### Genome

**Figure 1:**
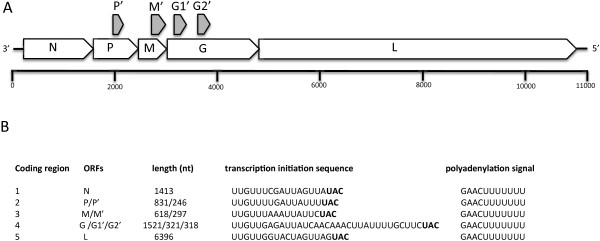
**Organization of the LITRV genome. A)** Schematic representation of LITRV ORFs. **B)** LITRV coding regions and corresponding transcription regulatory sequences.

**Figure 2:**
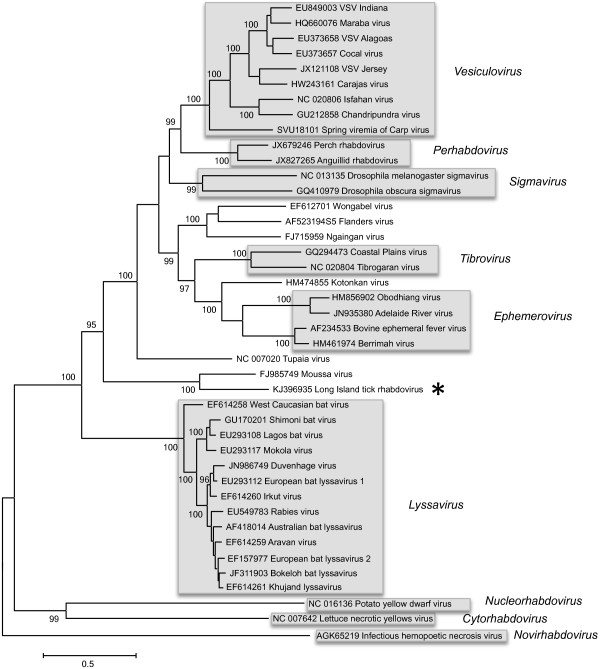
**Phylogeny of LITRV.** Maximum likelihood phylogenetic tree based on full length L protein sequences of currently recognized species of *Rhabdoviridae*. Gray boxes represent ICTV-accepted species within the genus. LITRV is indicated by *. Accession numbers are provided next to the viral names. Due to excessive divergence, only the type species for *Cytorhabdovirus*, *Novirhabdovirus* and *Nucleorhabdovirus* are included. The *Vesiculovirus* piry virus is not included due to limited available sequence.

The complete genome of LITRV comprises 11,176 nucleotides (nt), contains non-coding 3′ and 5′ sequences, and five main ORFs encoded in linear order (Figure 1A). Characteristic of rhabdoviruses, the coding regions are flanked by conserved sequences that likely serve as transcription initiation and transcription termination/polyadenylation signals. The polyadenylation signal consists of 3′- GAACUUUUUUU, which is followed by a 2 nt intergenic sequence and a putative transcription initiation sequence 3′-UUGUU(U/G)N(G/A/U)U (Figure 1B). Homology search of all five coding regions revealed that each ORF is most similar to the corresponding ORF of MOUV (Table 1). Both MOUV and LITRV cluster together and form a distinct phylogenetic clade within *Rhabdoviridae* (Figure 2). Similar to MOUV, only ORFs 1, 4 and 5, encoding the putative N, G and L proteins display homology to corresponding *Rhabdoviridae* proteins. ORFs 2 and 3, encoding the putative P and M proteins display no homology to any rhabdovirus proteins outside of MOUV. The LITRV genome also contains four alternative ORFs located within the P, M and G ORFs. These ORFs were designated P’, M’ and G1’ and G2’, and would encode proteins of 81, 98, 106 and 105 amino acids (aa), respectively, with no significant sequence identity with any other *Rhabdoviridae* protein by BLASTp analysis. Of the four ORFs, only P’ and G2’ have an initiation codon in suitable context for translation.

**Table 1:** Comparison of LITRV and MOUV ORFs

	**ORF**				
	**N**	**P**	**M**	**G**	**L**
LITRV	1413 nt	831 nt	618 nt	1521	6396
	470 aa (37.2%)*	276 aa (20.8%)	205 aa (28.2%)	506 aa (28.5%)	2131 aa (51.7%)
MOUV	1404 nt	870 nt	726 nt	1581	6428 nt
	467 aa	289 aa	241 aa	526 aa	2141 aa

*Indicates % amino acid identity of LITRV to MOUV.

As in all rhabdoviruses, the 3′ and 5′ terminal non-coding regions of LITRV contain partially complementary regions, particularly at the terminal ends. The 3′ leader sequence is 49 nt long while the length of the 5′ trailer sequence is 115 nt. The terminal portions of the non-coding sequences are highly conserved in both LITRV and MOUV with 13 out of 19 nt identical in both viruses.

### ORF analysis

The 1413 nt ORF1 is predicted to encode a nucleoprotein of 470 aa that is 38% similar to MOUV N and contains many conserved rhabdovirus N domains including the RNA binding motif SPYS (Table 1). ORF2, comprising 831 nt, and, in accordance with its position within the rhabdoviral genome, is predicted to encode a 276 aa phosphoprotein. This protein shares 22.3% identity with ORF2 of Moussa virus and is predicted to contain 17 serine, 6 threonine and 1 tyrosine potential phosphorylation sites (http://www.cbs.dtu.dk/services/NetPhos/). The 618 nt ORF3 is predicted to encode a 206 aa protein. The protein shares 29.4% identity with MOUV ORF3. No other known motifs or domains were recognized, although a polyproline region, consisting of 8 consecutive proline residues was identified at aa 43–50. ORF 4, comprising 1521 nt, is predicted to encode a 506 aa class I transmembrane glycoprotein with 29.4% aa identity to MOUV G protein. The protein sequence contains a predicted N terminal 18 aa signal peptidase cleavage site, a hydrophobic trans-membrane domain at positions aa 458–480 followed by a C terminal 26 aa tail. It also contains 5 potential N-glycosylation sites at aa positions 58, 345, 359, 387, 424 and contains all 12 cysteine residues conserved in other rhabdoviruses. The 6396 nt L ORF encodes the RNA-dependent RNA polymerase, that in LITRV is predicted to encode a 2131 aa protein. The LITRV L is 51.5% identical to MOUV and contains all the highly conserved residues of negative strand RNA polymerases [4].

To examine the presence of LITRV in ticks, we screened archived cDNA generated from 50 individual adult *A. americanum* by polymerase chain reaction. The ticks were collected in April of 2008 in the same location as the ticks from the current study. LITRV sequence was successfully amplified from one tick.

## Discussion

*Rhabdoviridae* is a large family that includes over 100 viruses classified together on the basis of genetic, serological or morphological analysis. The advent of high-throughput sequencing has resulted in genome characterization of many novel and archived rhabdoviruses revealing the vast diversity of this family [3,5-10]. In this study we present the first complete genomic sequence of a tick-associated rhabdovirus from the Western hemisphere. Although arthropods are frequently implicated as hosts of rhabdoviruses, thus far relatively few of these viruses have been associated with ticks [1,5,11]. We detected LITRV in *A. americanum,* a common tick species with a broad range spanning eastern and south-central states in the US. Along with *Ixodes scapularis* and *Dermacentor variablis, A. americanum* is the primary human-biting hard tick in the eastern part of the US and is implicated in transmission of *Ehrlichia* species, *Borrelia lonestari*, *Francisella tularensis*, and the Heartland virus [12,13].

Additional studies are needed to determine if *A. americanum* is the primary host for LITRV or if other arthropods, and particularly, other ticks, have a role in its enzoonotic transmission. While sequences were obtained form *A. americanum*, this does not definitively implicate *A. americanum* as a biological host for LITRV. Detection of microbial nucleic acids in organisms other than their presumed biological hosts has been reported and in a hematophagus organism these can represent nucleic acid remains of microbes acquired as part of a blood meal [14,15]. However, our detection of LITRV in *A. americanum* collected at the same site five years apart is consistent with a role for this tick species in the life cycle of this virus. Furthermore, deep sequencing analysis of multiple pools of *I. scapularis* and *D. variabilis* collected from the same location and did not identify any LITRV-like sequences in these two tick species. Our initial survey of LITRV in individual *A. americanum* suggests that the prevalence of this virus may be low in tick populations within the examined area. Molecular surveys analyzing different life stages of *A. americanum* and other tick species in broader geographical areas are necessary to establish the range and prevalence of LITRV.

Phylogenetically, LITRV forms a distinct clade with MOUV. MOUV was isolated from multiple mosquito pools in Cote d’Ivoire, suggesting that arthropods are the likely hosts to this clade of rhabdoviruses. LITRV and MOU share many genetic similarities, including identical polyadenylation and terminal regions, as well as similar conserved 3′ and 5′ termini sequences. The high amino acid divergence of LITRV and MOUV relative to other rhabdoviruses suggests that both viruses are unlikely to be associated with any of the current ascribed genera and likely represent a unique taxonomic group within *Rhabdoviridae*. We anticipate that continued surveillance of arthropod vectors may uncover other members of this clade.

## Conclusions

Using high-throughput sequencing analysis of tick viromes, we discovered a novel tick associated rhabdovirus. This virus, which we provisionally name Long Island tick rhabdovirus, may represent a novel species within family *Rhabdoviridae*.

## Materials and methods

### Nucleic acid extraction

Adult ticks were collected in Heckscher State Park (Suffolk County, NY) in April, 2013. Ticks were pooled and homogenized in 500 μl of phosphate buffered saline. Five tick pools were analyzed; one pool of *A. americanum* (N = 25), and two pools each (N = 30/pool) of *I. scapularis* and *D. variablis*. The homogenate was purified through 0.22 μM filter and treated with RNaseA and TurboDNase exonucleases. 250 μl of the filtrate was added to 750 μl of NucliSens buffer and total nucleic acid (TNA) was extracted with the EasyMag extraction platform (Biomerieux). TNA was eluted in 35 μl volume followed by DNase treatment.

### Unbiased high throughput sequencing

Total nucleic acid was subjected to first and second strand cDNA synthesis with Super Script III reverse transcriptase (Invitrogen) and Klenow Fragment (New England Biolabs), respectively. Ion Shear™ Plus Reagents Kit (Life Technologies) was used for double stranded cDNA fragmentation. Ion Xpress™ Adapters and unique Ion Xpress™ Barcodes (Life Technologies) were ligated to fragmented material by using the Ion Plus Fragment Library kit, which also contained reagents for amplification of barcoded libraries. Ion OneTouch™ 200 Template Kit v2 (Life Technologies) was used to bind barcoded libraries to Ion Sphere™ particles (ISPS). Emulsion PCR of DNA linked ISPS was performed on the Ion OneTouch™ 2 instrument (Life Technologies). Ion OneTouch™ ES instrument was used to isolate template-positive ISPS. Ion PGM™ Sequencing 200 Kit v2 (Life Technologies) was used for sequencing of templated ISPS which were loaded on the Ion 316™ Chip for further processing on the Ion Personal Genome Machine® (PGM™) System (Life Technologies).

The de-multiplexed reads were preprocessed by trimming primers and adaptors, length filtering, and masking of low complexity regions (WU-BLAST 2.0). The remaining reads were subjected to homology search using BLASTn against a host genome database. The host-subtracted reads were assembled using the Newbler assembler (454, v2.6). Contigs and singletons were then subjected to a homology search against the entire GenBank database using BLASTn and the viral GenBank database using BLASTx. Contigs and singletons with similarity to viral sequences from the BLASTx analysis were again subjected to a homology search against entire GenBank database to correct for biased e-values. For potential viral candidates, close relatives were used to identify low homology regions in the genome from BLASTx. Overall, out of approximately 190,000 sequence reads obtained by HTS, 96 reads with a mean length of 182 nt were unique to LITRV. Gaps were filled in by PCR using primers specific to the assembled sequence. The final sequence was verified by classical dideoxy sequencing using primers designed to generate overlapping PCR products. Genomic termini were obtained by 5′ and 3′ RACE kits (CloneTech). Genome assembly was performed with Geneious v 6.1. All phylogenetic trees were constructed with Mega 5.2 software.

### Tick screening

To assess the presence of Long Island tick rhabdovirus in ticks, we used cDNA generated from adult *A. americanum* ticks collected in April, 2008 at the same location. Tick cDNA was screened by PCR with primers 5′-GGGACGATGCTCTAGTCACG-3′ (fwd), and 5′-TTTGTCTGTGAGGTCGGACG-3′ (rev) targeting a 299 bp fragment of the N gene. PCR products were assessed by gel electrophoresis and sequenced to confirm that they represent Long Island tick rhabdovirus.

The complete genome sequence of LITRV was deposited in Genebank under accession number KJ396935.

## Competing interests

The authors declare that they have no competing interests.

## Authors’ contributions

RT and WIL conceived the study, analyzed data and wrote the manuscript. SS and MSL performed all assays. KJ performed all bioinformatics analysis. All authors read and approved the final manuscript.
